# Tissue Concentrations of Zinc, Iron, Copper, and Magnesium During the Phases of Full Thickness Wound Healing in a Rodent Model

**DOI:** 10.1007/s12011-018-1600-y

**Published:** 2018-12-14

**Authors:** Vincent Coger, Nina Million, Christoph Rehbock, Bernd Sures, Milen Nachev, Stephan Barcikowski, Nina Wistuba, Sarah Strauß, Peter M. Vogt

**Affiliations:** 1 Department of Plastic, Aesthetic, Hand and Reconstructive Surgery, Hannover Medical School, Hannover, Germany; 2 Technical Chemistry I and Center for Nanointegration Duisburg-Essen, University of Duisburg-Essen, Duisburg, Germany; 3 Aquatic Ecology and Centre for Water and Environmental Research (ZWU), University of Duisburg-Essen, Essen, Germany

**Keywords:** Skin wound healing, Metal, Zinc, Iron, Copper, Magnesium

## Abstract

Wound healing is a complex orchestration of processes involving cell proliferation, migration, differentiation, anabolism, and catabolism in order to restore skin continuity. Within these processes, elements such as metallic ions are involved due to their implications in cell behavior and enzymatic activity regulation. This study analyzed the kinetics of zinc, iron, copper and magnesium concentrations in a full thickness open wound rat model over 14 days. We made wounds with a diameter of 6 mm on the back of Lewis rats and let them heal naturally prior to analysis by histology and inductively coupled plasma mass spectrometry analysis. Histological and immunofluorescence analysis confirmed an inflammation phase until 7 days, epithelial proliferation phase from 16 h to 10 days, and remodeling phase from 7 days onward. These defined phases were correlated with the measured metal element kinetics. Zinc concentrations showed an inverted parabolic progression between 30.4 and a maximum of 39.9 μg/g dry weight. Magnesium values had a similar pattern between 283 and 499 μg/g dry weight. Copper concentrations, on the other hand, followed an inverted sigmoid trend with a decrease from 9.8 to 1.5 μg/g dry weight. Iron had a slight decrease in concentration for 24 h followed by an increase to a maximum of 466 μg/g dry weight. In conclusion, zinc, iron, and copper, even though differing in their total mass within the wound, exhibited concentration curve transitions at day 3. Interestingly, this time point correlates with the maximum proliferating keratinocyte rate during the proliferation phase.

**Electronic supplementary material:**

The online version of this article (10.1007/s12011-018-1600-y) contains supplementary material, which is available to authorized users.

## Introduction

Open wounds create a major impact on the healthcare system because of the financial cost of the medical treatment, pain for the patient, and emotional cost from reduced self-esteem associated with scars. Wound healing processes have been studied for centuries. Due to the complexity of these processes, several fields of biology and medical science are involved in understanding the physiological development, cellular processes, and enzymatic machineries that contribute to the reestablishment of skin integrity. In addition to currently studied models, a quantitative determination of the essential elements Mg, Cu, Zn, and Fe in the wound during the skin restoration process may improve the understanding of wound healing and provide a basis for further research into this topic.

Wound healing consists of three overlapping phases, which include five processes: inflammation with hemostasis and inflammatory processes, proliferation with granulation and repidermalization processes, and finally the remodeling phase [1]. These phases are interdependent with each other and overlap in time. The duration of each wound healing phase depends on several factors [2–4]: wound type and size, age, physical condition, comorbidities, location, and wound treatment. The importance of metals has been highlighted by many studies regarding metal deficiencies and the ability to reestablish wound healing by metal oral supplementation [5]. Local supplementation was shown to induce several beneficial effects including acceleration of healing and strengthening of the cicatricial tissue [6, 7].

In the whole organism, as well as in single cells, metallostasis is precisely regulated. The dynamic relationship among the different metal elements is extremely complex and two or more of these metals can be competitors and bind to the same protein, inducing direct or indirect regulation of its activity. The binding affinities are responsible for fine regulation of enzymatic or other protein activities and ion availability. For example, copper and zinc are both susceptible to bind to metallothionein, a metal binding protein that regulates the intracellular metal availability. Zinc induces metallothionein gene transcription which was shown to reduce the half-life of the protein in comparison to copper [8].

The abilities of numerous metal ions to modify cell metabolism as well as phenotype are variable. For instance, zinc induces keratinocytes differentiation [9], magnesium supports their adhesion to laminin [10], and iron and copper induce fibroblast expression of matrix metalloproteinase-1 (MMP-1) [11, 12]. Although many studies try to use metal ions to enhance wound healing, the basic concentrations of these elements during different phases of wound healing remains a niche research field supported by scattered studies [13].

Our study focuses on four enzyme-associated elements that, based on their abundance in the literature and their success in clinical studies, are imperative for the proper course of wound healing: zinc, iron, magnesium, and copper. We describe the kinetics of these elements in the phases of natural healing of a full thickness skin wound model in rats. We characterized the cicatrization processes histologically, which allows us to correlate individual metal concentrations at different time points with the different phases of wound healing (Table 1).

**Table 1:**
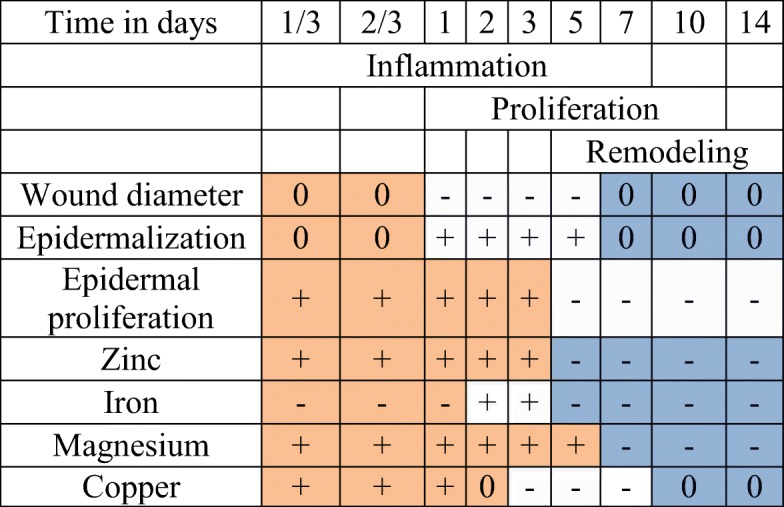
Evolution of the kinetics in phases of wound healing. Results are represented as constant (0), decrease (−), or increase (+) in comparison to the previous time point

## Materials and Methods

### Animal Experiments

All experiments were performed according to the German animal protection act and approved by the Institutional Committee on Use and Care of Animals of Hanover Medical School and the Lower Saxony State Board on Care and Use of Animals (LAVES; Oldenburg, Lower Saxony, TV 12/0993). Twenty-seven male Lewis rats (Charles River; LEW/Crl) with an age of 8 weeks were housed under specific pathogen-free condition with 12-h day-night cycle, with food and water ad libitum. Animals were randomly divided into nine time-groups (*n* = 3): 8 and 16 h and 1, 2, 3, 5, 7, 10, and 14 days.

General anesthesia was induced in a narcosis chamber (Euthanex Corp.; P.O. Box 3544) ventilated at 2.5 l/min oxygen flow and 5% Isoflurane (Baxter; HDG9623). Once asleep, masks were placed on the rats and the Isoflurane concentration was diminished to 3%. The dorsal area was shaved and disinfected with Kodan Tinktur forte (Schültke; 25393-A). Novaminsulfon (Ratiopharm; 6882768) was administered subcutaneously (200 mg/kg of body weight) pre-operatively. Six whole skin biopsies (6-mm diameter) were taken with a biopsy punch (6-mm biopsy punch; Stiefel; 05.SF004.11/08) and preserved as control sample. Post-operative analgesia was maintained by dilution of 3.4 ml Novalgin (Ratiopharm; 3530394) in 500 ml of drinking water. Wounds were left and uncovered and air-exposed for the entire time to monitor the native healing process. The location of the wound prevented the rats from accessing the wounds and reopening them. Rats were housed individually to prevent cannibalization of the wounds by others. Wound contamination was reduced by replacing wood litter by dust-free wipes in sufficient amount for the rats to hide underneath. At the predefined time points, rats were anesthetized in the same manner as previously described and euthanized by cervical dislocation. To avoid contamination by surrounding bleeding, the whole dorsal skin was prepared prior to the wounds excision with 8-mm biopsy punches (8-mm biopsy punch; Stiefel; 05.SF004.11/08) including the scab. Three of the samples were fixed in 4% paraformaldehyde phosphate-buffered saline solution (0.1 M; pH = 7.4) for histological analyses, and the other three were shock-frozen in liquid nitrogen for inductively coupled plasma mass spectrometry (ICP-MS) measurements.

### Human Skin

The use of human skin tissue from plastic surgery patients undergoing reduction mammoplasty was approved by the ethical committee of Hannover Medical School (ref.no. 1055–2011). Patients (women; *n* = 3; 44 years old ± 17) gave their written consent for tissue donation. Human skin samples were submitted to the same protocol as described for the animal experiments.

### Histology

After 7 days of fixation in 4% paraformaldehyde phosphate-buffered saline solution (0.1 M; pH = 7.4), samples were washed with tap water for at least 3 h and dehydrated before paraffin embedding in a STP-120 (Mikrom) according to the previously described protocol [14]. The samples were impregnated with ethanol (Chemsolute; 2294.9010) at increasing concentrations from 50% to 99.9% (*V*/*V*), then with pure xylol (Roth; CN80.2), and finally with paraplast X-tra (Leica; 39603002). Samples were completely cut with a microtome (Mikrom) perpendicularly to the epidermis from side-to-side into 6-μm-thick slices. The center of the wound was determined as the median of the slices regarding the wound margins observed by light microscopy.

Hematoxylin-eosin (H&E) staining was performed for light microscopy analysis as previously described [14]. After rehydration of the thin cut with xylol, ethanol, and ultra-pure water, samples were stained 5 min in hematoxylin (Roth; T865.3), followed by rinsing 10 min in running tap water, then stained 5 min with eosin-G (Roth; X883.2), and quickly dehydrated prior to embedding under a coverslide with Roti-HistoFix II (Roth; T160.1). Light microscopy was performed with a Keyence BioZero with a × 4 objective and set at × 8 optical magnification. The entire wounds were documented for analysis.

We determined the wound width by measuring from merge to merge, and the epidermis outgrowth over the wound in millimeters by light microscopy histomorphometry of the H&E staining. The reepithelialization was calculated by setting epidermis growth as percentage of the wound width.

Immunofluorescence detection of Ki-67 was performed to visualize proliferating keratinocytes, coupled with nucleus DAPI staining (Vector Laboratories; H1200). To do this, slides were deparaffinized and rehydrated as described above. After an incubation for 25 min at 95 °C in Antigen Unmasking Solution (Vector Laboratories; H-3300), the tissue was permeated by incubation in tris-buffer-saline (TBS) at pH = 7.8 containing 0.3% Triton-× 100 (Sigma; 9002-93-1) for 5 min. Nonspecific reactions were blocked with TBS containing 2% of bovine serum albumin (BSA) for 1 h. The primary antibody, anti-Ki-67 (Thermo Scientific; Clone SP6, rabbit monoclonal antibody; RM-9106-S0) 1:100 diluted in TBS with 1% BSA, was incubated overnight at 4 °C in a wet chamber. After rinsing with TBS, a biotinylated secondary antibody (polyclonal goat anti-rabbit immunoglobulins/Biotinylated Dako; E-0432) diluted 1:200 in TBS was applied at room temperature for 90 min, rinsed with TBS, and incubated with Vectastain ABC Kit (Vector laboratories; PK-4000) 1 h at room temperature according to the manufacturer’s instructions and followed by 5-min room temperature incubation under a Cy3 Tyramide solution (Perkin Elmer; SAT704A001EA). After washing, slides were covered with Vectashield Mounting Medium with DAPI (Vector Laboratories; H1200) under cover slips.

Fluorescence microscopy was accomplished with a Zeiss AxioVert 200M with a × 20 objective. The healthy epidermis of the wound edge was documented. This was determined by a decrease in density of the extracellular matrix (ECM), as well as the presence of dermal appendages like hair follicles. Ki-67-positive keratinocytes in healthy epidermis were counted and normalized to the measured length of the same studied epidermis. The results were presented as number of positive KI-67 cells per millimeter in length of epidermis (*U*) in function of time. Image analysis was performed with Cell-D software (Olympus) after the proper calibration of the pictures.

### Inductively Coupled Plasma Mass Spectrometry

After heat dehydration (3 days in dry chamber at 80 °C), samples were weighed and digested using a microwave-accelerated reaction system (CEM, Mars 5). Each sample was placed in a fluoropolymer vessel (XP-1500 plus) and mixed with 1.3 ml nitric acid (65%, Suprapure, Merck) and 2.5 ml hydrogen peroxide (30%, Suprapure, Merck). After digestion at 600 W, each sample solution was poured completely into a 5-ml volumetric glass flask and brought up to volume using double distilled water. The samples were stored at room temperature until measurement.

Inductively coupled plasma mass spectrometry (ICP-MS) was used to determine the metal concentrations [15]. These measurements were carried out using a quadrupole ICP-MS system (Perkin Elmer, Elan 6000) with an auto sampler system (Perkin Elmer, AS-90). The instrument operated at 1000-W plasma power, 14 l/min plasma gas flow, 0.95 l/min nebulizer gas flow, and a sample flow rate of 1 ml/min regulated by a peristaltic pump. Between each measurement, the wash time with 1% HNO_3_ (Suprapure, Merck) was set to 10 s to avoid contaminations. The accuracy and stability of the measurements was controlled by a multi-element standard solution (ICP multielement standard IV solution, Merck) once every 10 samples. Samples were diluted 1:10 with an internal standard solution, consisting of 1% HNO_3_ containing 10 μg/l yttrium and 10 μg/l thulium. A calibration was carried out with a series of 11 dilutions of a multi-element standard solution (ICP multielement standard solution, Merck). With this calibration, the concentrations of the analytes were calculated using the corresponding regression lines with a correlation factor of ≥ 0.999.

To validate the analytical procedure, we determined the concentrations of iron, copper, and zinc in two different reference materials, DORM-3 (National Research Council (NRC) Canada) and Oyster tissue (National Institute of Standards and Technology (NIST) Gaithersburg (USA)). Additionally, we prepared five samples for ICP-MS. Each of them consisted of 25 mg of healthy rat skin, mixed together with different concentrations of copper and zinc varying from 0 to 10 ppb (Copper standard solution and zinc standard solution, Merck). After preparation by digestion and dilution as mentioned before, all samples were measured by ICP-MS.

### Statistical Analysis

Each group data resulted from the mean of 3 rats (*n* = 3). Each rat’s values were the average of its wounds values (*n* = 3 for ICP-MS, Ki-67, and H-E).

Data were statistically analyzed with IBM SPSS Statistics to calculate means, standard deviation of the mean, analysis of variance (ANOVA), and Bonferroni’s post hoc correction for significance determination at a 95% confidence level. We used a Pearson correlation analysis between the different kinetics obtained.

## Results

**Fig. 1:**
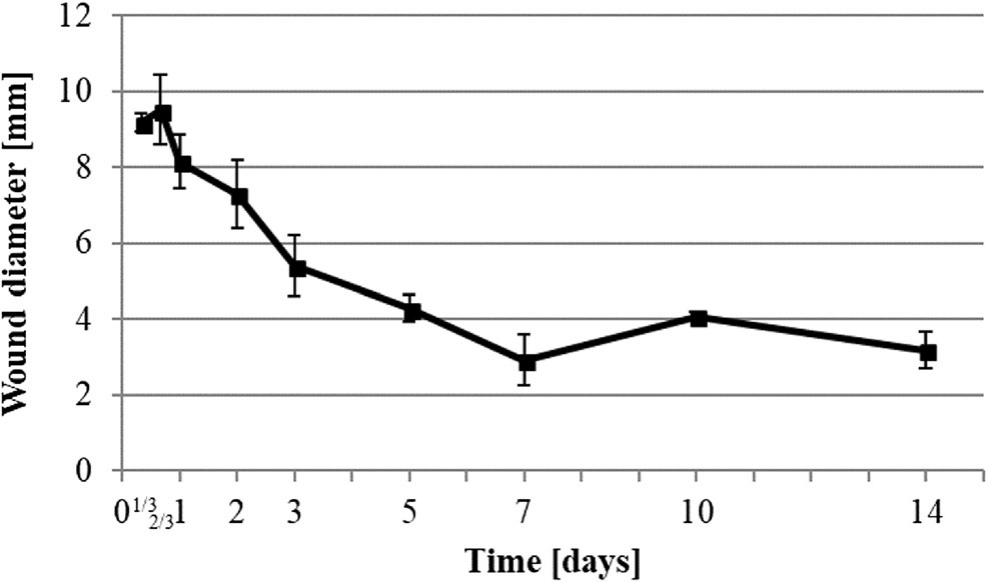
Histologic measurement of wound diameter development. Data were calculated by measurements of wound margins of H&E staining of 6-μm thin cut of wounds taken at the median part of the wound ± SD (*n* = 3 rats in each group; one rats data is the mean of 3 wounds)

**Fig. 2:**
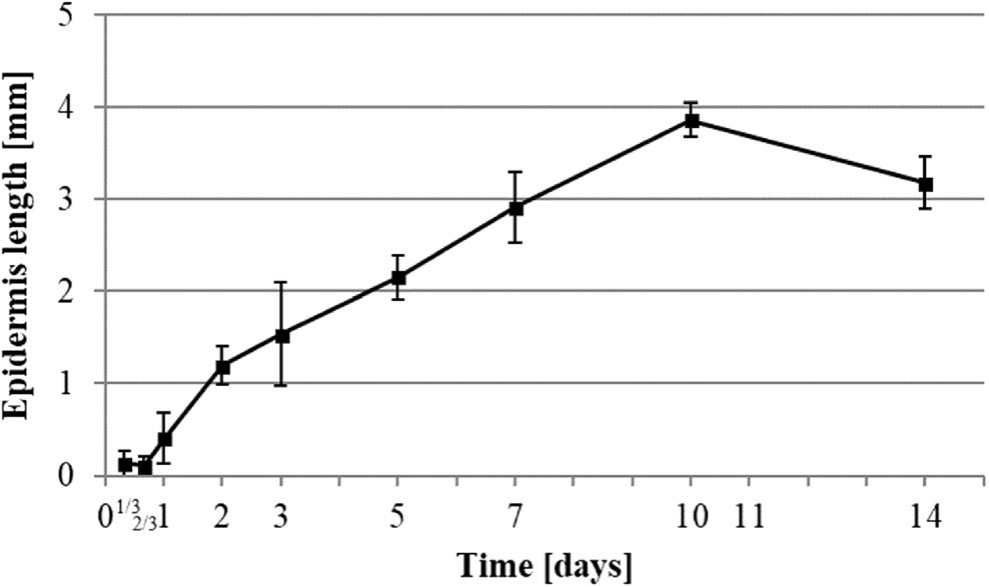
Histologic measurement of epidermis development. Data were calculated by measurement of growing epidermis from the margins of H&E staining of 6-μm thin cut of wounds taken at the median part of the wound ± SD (*n* = 3 rats in each group; one rats data is the mean of 3 wounds)

**Fig. 3:**
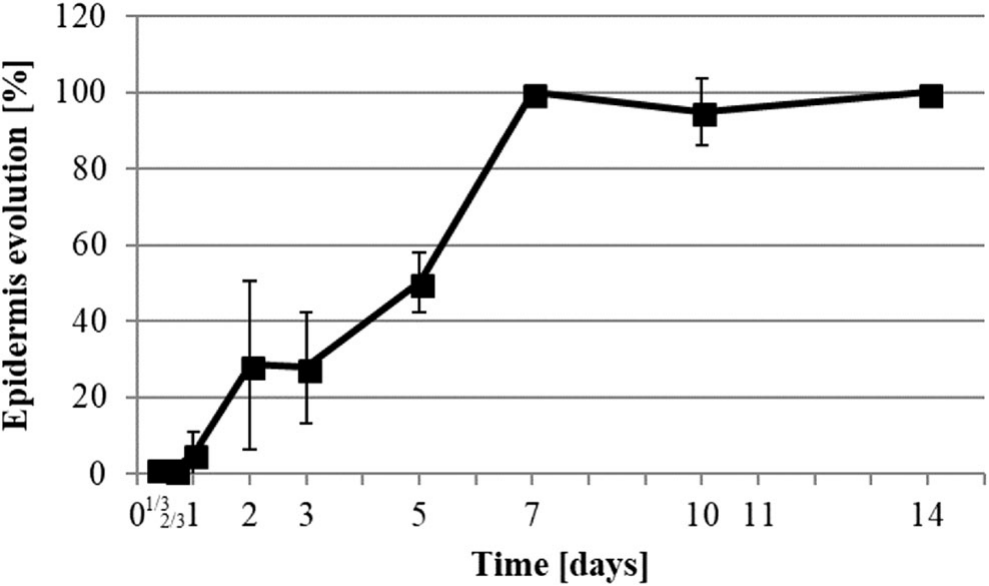
Histologic measurement of reepithelialization relative to the wound diameter. Data were calculated by measurement of growing epidermis from the margins normalized to the wound diameter of H&E staining of 6-μm thin cut of wounds taken at the median part of the wound ± SD (*n* = 3 rats in each group; one rats data is the mean of 3 wounds)

To identify the wound healing phases of the samples, we performed H&E staining. The wound diameters (Fig. 1) as well as the epidermis progression onto the wound (Figs. 2 and 3) were measured. Despite of the use of a 6-mm diameter biopsy punch for the wounding, the embedding and microtomic processing led to an optical appearance which was larger than the initially induced wound diameter (Fig. 1). The wounds remained stable in diameter for 16-h post-operation with a diameter of 9 mm ± 0.5 mm. Afterwards, the diameter of the wound rapidly decreased until day 7 (2.9 mm ± 0.7). A slight relaxation to 4.1 mm ± 0.1 could be observed at day 10 before the diameter reached 3.2 mm ± 0.5 at day 14.

H&E staining visualized the reepithelialization process of the wound (Figs. 2 and 3), starting between 16 and 24 h after wounding. Once the reepithelialization started, the process continued at a rate of 0.33 mm per day until day 7 by which the epidermis covered 100% of the wound (Fig. 3). Due to the above-mentioned relaxation of the wound diameter at the 10th day (Fig. 1), we could still measure an increase in the length of the epidermis in the wound at day 10 (Fig. 2), even if it had fully covered the wound site from day 7.

**Fig. 4:**
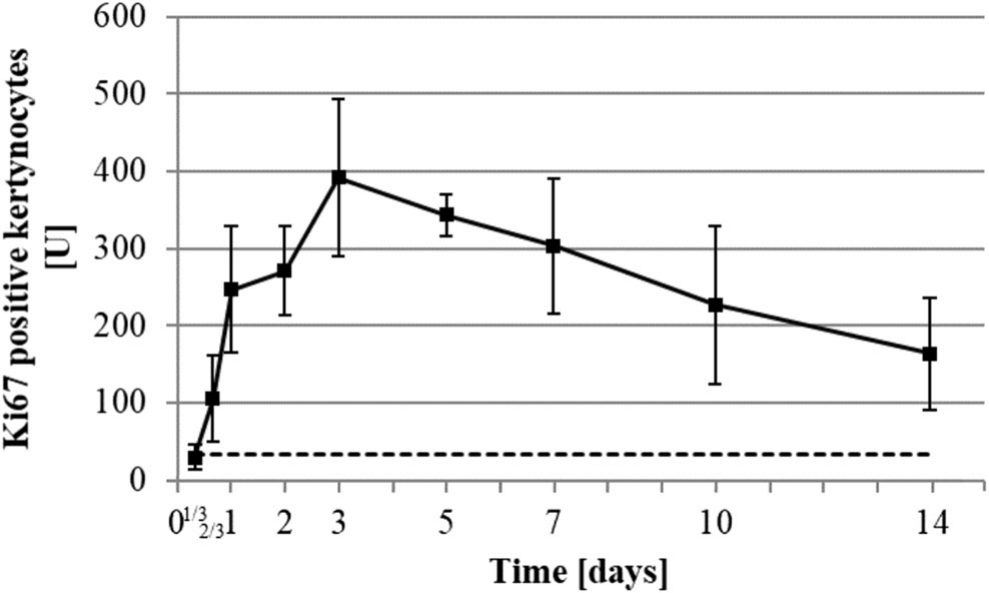
Immunofluorescence measurement of number of Ki-67-positive keratinocytes at the wound margins in time in relation to the observed epidermis length. Dot line represents healthy skin baseline (0.03 *U* ± 0.02). All data were displayed as mean ± SD of three rats (*n* = 3 rats in each group; one rat’s data is the mean of 3 wounds)

We also performed immune-fluorescence staining against Ki-67 and counted the number of positive Ki-67 keratinocytes per millimeter of epidermis (*U*) in the wound margin (85 μm ± 27) (Fig. 4). Keratinocyte proliferation increased from the 16th hour up to a maximum of 30 *U* ± 15 on the 3rd day. Then the relative number of proliferative keratinocytes decreased slowly, but its level at the 14th day was still higher compared to healthy skin (163 ± 72 versus 33 ± 21 *U P* = 0.02).

After acidic degradation of the dried wound samples (mean dry weight of the samples 30.7 ± 4.0 mg), metal elements were quantified by ICP-MS. As a baseline, we determined the concentration of those elements in the healthy skin that we excised with biopsies (mean dry weight of the samples 14.3 ± 3.1 mg) in order to set the wounds (Table 2). We also performed ICP-MS on human skin obtained from reduction mammoplasty (mean dry weight of the samples 33.0 ± 14 mg). We noticed two concentration ranges: magnesium and iron which were higher than 100 μg/g of dry weight and zinc and copper that were below 50 μg/g of dry weight.

**Table 2:** Concentration baselines in rat and human healthy skin. ICP-MS results of the mean of at least 3 samples ± SD. * [16]

Baselines	Magnesium	Iron	Copper	Zinc
Human	N/A	149.9 ± 43.5 μg/g	2.8 ± 0.11 μg/g	28.9 ± 2.98 μg/g
	(10 to 150 μg/g*)			
Rat	331.7 ± 24.7 μg/g	76.0 ± 40.9 μg/g	9.1 ± 4.14 μg/g	39.1 ± 7.32 μg/g

**Fig. 5:**
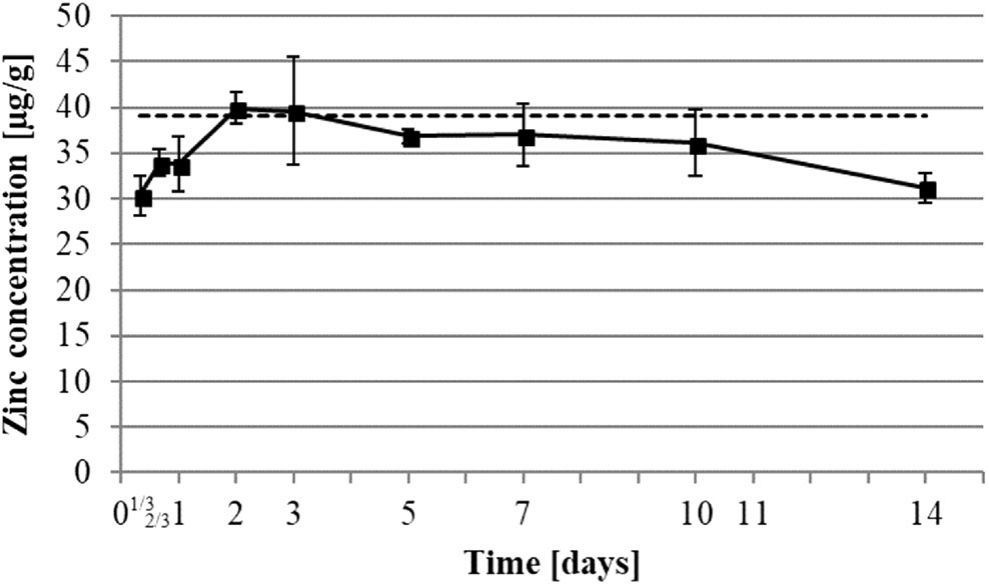
Development of zinc concentration in healing wound. Zinc element was determined by ICP-MS. Dot line represents healthy skin baseline. All data were displayed as mean ± SD of three rats (*n* = 3 rats in each group; one rat’s data is the mean of 3 wounds)

Healthy skin of rats showed a baseline level of zinc of 39.1 ± 7.3 μg/g of dry weight (Table 2 and Fig. 5). In the wounds, level of zinc was lower with 30.4 ± 2.1 μg/g at 8 h, reached the baseline level of 39.9 ± 1.7 μg/g of dry weight between days 2 and 3, and then progressively decreased to 31.2 ± 1.6 μg/g of dry weight until day 14 post-operation (Fig. 5).

**Fig. 6:**
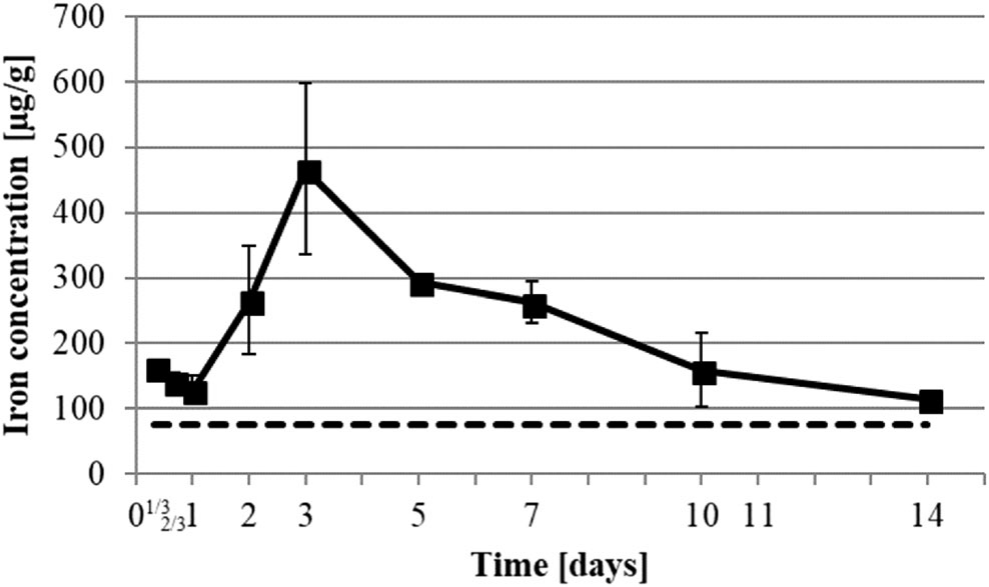
Development of iron concentration in healing wound. Iron element was determined by ICP-MS. Dot line represents healthy skin baseline. All data were displayed as mean ± SD of three rats (*n* = 3 rats in each group; one rat’s data is the mean of 3 wounds)

**Fig. 7:**
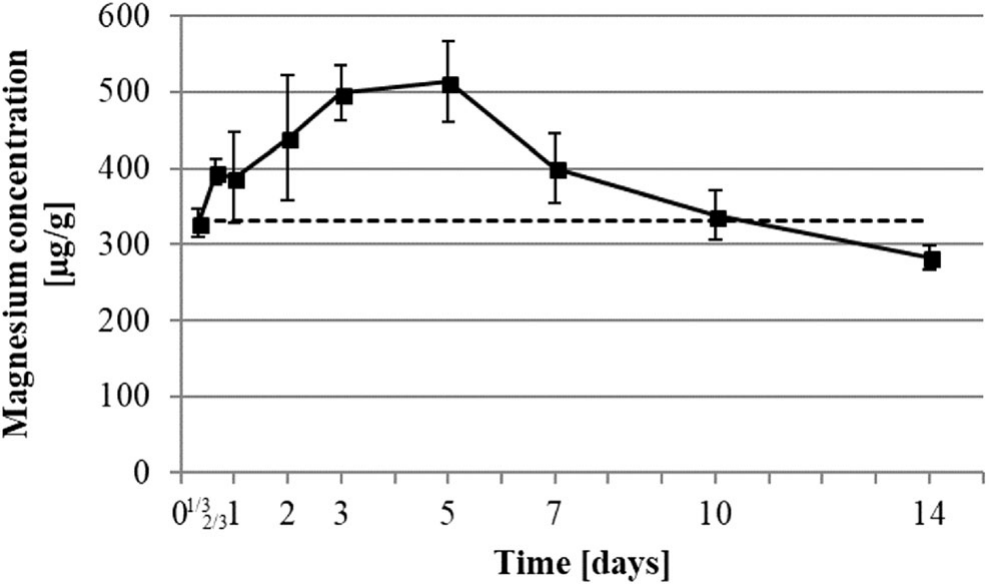
Development of magnesium concentration in healing wound. Magnesium element was determined by ICP-MS. Dot line represents healthy skin baseline. All data were displayed as mean ± SD of three rats (*n* = 3 rats in each group; one rat’s data is the mean of 3 wounds)

The iron concentration (Fig. 6) dropped down in the first 24 h from 164 ± 12 to 129 ± 22 μg/g and rapidly increased to 467 ± 131 μg/g on day 3. Then it decreased slowly to reach 116 ± 9 μg/g on day 14. The magnesium concentration (Fig. 7) slowly increased from a baseline of 332 ± 25 to 513 ± 53 μg/g of dry weight within the first 5 days followed by a decrease to 283 ± 16 μg/g on day 14.

**Fig. 8:**
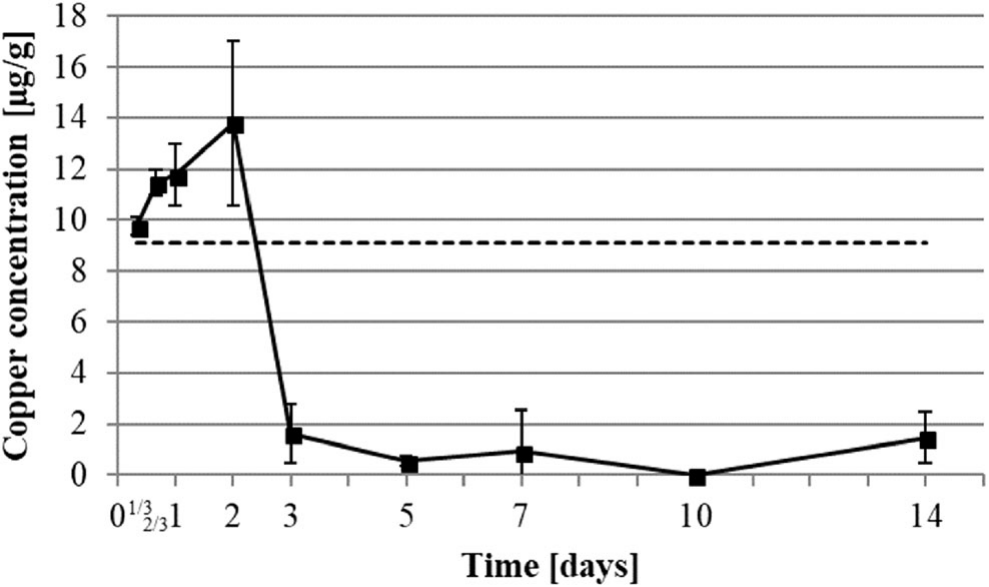
Development of copper concentration in healing wound. Copper element was determined by ICP-MS. Dot line represents healthy skin baseline. All data were displayed as mean ± SD of three rats (*n* = 3 rats in each group; one rat’s data is the mean of 3 wounds)

The temporal course of copper concentration (Fig. 8) showed an increase from 9.1 ± 4.1 to 13.8 ± 3.2 μg/g in the first 2 days before a drastic reduction below 2 μg/g between days 3 and 5 (1.6 ± 1.1 and 0.5 ± 0.2 μg/g respectively). It then remained stable under 1.5 μg/g until the end of the experiment.

We searched for correlation between the kinetics by calculating the Pearson correlation coefficient (PCC). Due to the biological variance, the kinetics were considered to correlate when the PCC was less than or equal to − 0.8 or greater than or equal to 0.8. The kinetics of the different metal element concentrations, the wound diameter, the wound reepithelialization, and the number of positive-Ki-67 keratinocytes per millimeter of epidermis were analyzed for correlations. We found a statistically relevant inverted correlation between the wound diameter and the reepithelialization of − 0.88 (*P* < 0.001). Of the metal contents, only copper showed significant correlations with the wound diameter and the reepithelialization of 0.86 and − 0.83 respectively (*P* < 0.001). As the percentage of reepithelialization results from the normalization of the length of reepithelialization relative to the wound diameter, correlation between these parameters was not calculated.

Rat skin magnesium content was measured to be 332 ± 25 μg/g of dry weight, whereas published literature states it between 10 and 150 μg/g in human skin (magnesium content for human skin not measured in our study) [16]. Iron level in human and rat skin was measured respectively at 150 ± 44 and 76.0 ± 41 μg/g of dry weight, but were not significantly different (*P* = 0.46). Copper was measured at 2.8 ± 0.1 and 9.1 ± 4.1 μg/g of dry weight in human and rat skin, respectively. Zinc was measured at 28.9 ± 3.0 and 39.1 ± 7.3 μg/g of dry weight (*P* = 0.23) in human and rat skin, respectively. The difference between human and rat skin content was significant for copper concentration (*P* < 0.01) (Table 2).

## Discussion

### Inflammation

The inflammation phase aims to avoid physiological disturbances by first reestablishing hemostasis, counteracting any pathogens, and starting debridement of the wound bed. [1]. As skin lesions trigger wound healing, we assume the inflammation phase to start at the very beginning of the experiment. According to published literature, we estimated the inflammation phase would decline with the progression of reepithelialization [17]. The inflammation takes place at the open area of the wound between the keratinocyte migration front that reduces the exposed area from day 1 until day 7 (Fig. 2).

### Proliferation

The proliferation phase is composed of two main processes: epithelialization involving keratinocyte proliferation and migration on top of the wound bed, and granulation involving fibroblasts that proliferate, migrate, and synthetize ECM constituents.

### Granulation

Fibroblasts from the surrounding dermis and circulating fibroblasts are induced to migrate into the fibrin clot. Once there, they proliferate and synthetize type III collagen and fibronectin [18, 19]. This modification enables cell migration of keratinocytes onto the granulation tissue [20]. The beginning of the granulation tissue growth also marks the beginning of the proliferation phase. Keratinocytes in our trial started to migrate 24 h after wounding (Fig. 3), so this time can be considered as the starting point for granulation.

### Reepithelialization

The final step of wound healing restores the impermeability of the skin barrier by reepithelialization. The pattern of keratinocytes is a reliable observation that can be used to define the evolution of wound healing and its phases [1]. Therefore, we defined the beginning of the proliferation phase at the point at which they began to migrate onto the wound at 1-day post-operation (Figs. 2 and 3).

The higher number of proliferating keratinocytes per millimeters in comparison of the literature [21] may be the result of the difference in the species studied, as well as the narrower epidermis length we analyzed at the margin of the rat’s wounds.

### Remodeling

The remodeling phase aims to replace the granulation tissue with healthier skin. This process takes a long time in comparison with the other phases. The resulting scar tends to match physical and physiological properties of the original skin. This phase occurs when the epidermis is fully restored [1, 13, 22]. In our results, full reepithelialization occurred on the 7th day post-operation. Therefore, we can assume the remodeling phase starts at this time.

### Metals in the Phases of Wound Healing

Significant difference was observed between the copper baseline values determined in rat and human healthy skin. This can be due to the differences in several parameters, aside of the species considerations: the disparity in dermal appendages including the denser hairiness of the rats; the human skin was around two times thicker than those of rats (data not shown); the location of the human samples was thoracic; whereas rat’s biopsies were taken from the dorsum; in contrary to the rats used for our analysis, which have common genetic background and diet, human samples are subject to high variation. These differences have important implications for the common practice of scaling from rodents to humans but do not invalidate the kinetics we determined as the wound healing processes follow the same schema in the mammalian reign with minor variations.

### Zinc

Zinc is strongly related to enzymatic reactions and is described as cofactor of more than 300 enzymes including the MMP family and transcription factors. MMP is a major proteinase family that is responsible for enzymatic digestion of ECM constituents [23, 24]. The decrease of zinc content analyzed for the first time (Fig. 5) cannot be due to substance loss as we do not see this pattern in other metal concentrations (Figs. 6, 7, and 8). Instead, we hypothesize a reduced catabolism in the first steps of wound healing as ECM enzymatic digestion occurs in a nonspecific manner during the inflammation phase. During the first phase, temporary ECM has to be secreted and self-assembled. Later, during the proliferation phase, the replacement ECM is a result of protein synthesis and secretion. At this stage of wound healing, degradation of the ECM is more specific and replaces fibronectin with type III collagen. The rise of zinc concentration from 8 h to 3 days (Fig. 5) may induce the keratinocyte proliferation during the same time, as it has been shown that keratinocyte proliferation and differentiation are controlled by zinc [25, 26].

### Iron

Iron, aside of being the active center of hemoglobin, is actively implicated in cell metabolism, cell respiration, DNA synthesis, cell cycle control, and apoptosis triggering [27]. Our results showed enhanced iron concentrations during the whole experiment in comparison with healthy skin baseline (Table 2 and Fig. 6). Only a slight initial decrease from 8 h to 1 day was observed (Fig. 6). It is possible that the high level at the start of the experiment resulted from the bleeding at wound sites followed by phagocytosis and debridement of erythrocytes trapped in the clot. In the early stage of healing, phagocytosis of erythrocytes by macrophages leads to an elevation of their intracellular iron concentration. This induces their activation into pro-inflammatory macrophages (M1). M1 stimulate proliferation and migration of other cell types while retarding their differentiation [28]. Iron is also a regulator of the inflammatory response induced by epithelial cells through the secretion of lactoferrin [29]. Afterwards, iron concentration increased and reached a peak at day 3 (Fig. 6), which was the time we determined to be the peak of proliferation phase. Since iron is the active center of pro-collagen lysyl-hydroxylases, it is actively involved in the maturation of pro-collagen into a stabilized collagen triple helix [30]. Therefore, the high levels of iron during the mid-proliferation phase may participate in cell metabolism for proliferation and migration, as well as the anabolism of collagen. It was also noted that during the proliferation phase, neovascularization occurs, forming new blood vessels into the wound and thus reintroducing the granulation tissue to blood and iron that constitutes hemoglobin [4]. This can be correlated with the iron concentration increase observed from day 1 to day 3 (Fig. 6).

### Magnesium

Several studies implicated magnesium in various cell behaviors like proliferation, differentiation, and migration [27, 31], which are essential for wound healing processes. Also, extracellular levels of magnesium were proven to have direct effect on inflammation process [32]. In our model, the level of magnesium increased continuously from the skin baseline from the beginning of the experiment until the 5th day (Fig. 7). This increased concentration can be attributed to increased cell metabolism, which is needed during the proliferation phase. A peak was observed 5 days after wounding (Fig. 7). This corresponds to the peak of the proliferation phase between 24 h and 7 days. As previously described, the formation of cell-dense populations by rapid proliferation and deposition of collagen and fibronectin occurs during this phase. After this time, the proliferation phase decreases as the remodeling phase starts, and the magnesium level drops down. Energy-consuming cell behaviors like proliferation, differentiation, and migration are no longer required. The synthesis of definitive type I collagen and rearrangement of the ECM remains, which can occur without the required urgency of repair of a wounded site [1].

### Copper

Several studies underlined the ability of copper to enhance, ease, or reestablish healing of wounds by enhancing cell metabolism and proliferation [6]. A key regulator of copper intracellular metallostasis, the adenosine triphosphatase 7A (ATP-7A), is induced by platelet-derived growth factor (PDGF) released by the activated platelets [33]. Therefore, the observed initial augmentation of copper concentrations (Table 2 and Fig. 8) may result from an induced recruitment of copper at the wound site triggered by platelet degranulation. At the beginning of the proliferation phase, angiogenesis is strongly induced [1]. Intracellular copper concentration was shown to be a prerequisite for enabling angiogenesis induction via hypoxia-inducible factor-1 (HIF-1) activation of the VEGF transcription [6]. The increase in copper concentration we observed from the beginning of the experiment to day 2 (Fig. 8) might result in the neovascularization process as the granulation tissue begins to form. The kinetics of copper were slightly different compared to previous studies conducted by Lansdown et Al. [13], in which copper concentrations increased from healthy skin baseline to rapidly reach this level within 2 days and remained stable along the 10 days of experimentation. This might result from the differences in wounding design, as Lansdown et Al. [13] performed incisional full thickness wound sutured with no tissue loss, in contrary to our wound model. As the proliferation phase occurred from day 1 to 7, so did the granulation tissue neovascularization, and the reduction of copper concentration may be part of the slowing down of angiogenesis. It also seems to be combined with regulations that cause a rise of the zinc concentration.

Zinc and copper kinetics are interdependent due to their respective binding ability to the same proteins. This competition induces fine regulation of metal levels and, as the presence of copper is known to reduce zinc availability, we can hypothesize that the decrease in copper content may result from the elevation of zinc concentration and contributes to higher zinc bioavailability [8]. The in situ decrease in copper concentration (Fig. 8) between days 2 and 14 post-operatively could contribute to zinc availability for cell metabolism, migration, and proliferation, as well as ECM synthesis and digestion.

On the other hand, the profile of copper kinetics differs radically. In comparison to the healthy skin base, after slightly rising, it drops down after day 2 and ends at a tenth of the healthy skin concentration on day 7 (Fig. 8). This decrease correlates with the reepithelialization process which leads the evolution of the proliferation phase [1], so copper seems to play an important part in the early stage of wound healing and inflammation [34]. This can also result from the controlled environment that reduces the infection possibility and thus, the host inflammatory response. Moreover, the copper concentration obtained at day 14 post-operation (Fig. 8) leads us to the assumption that the wounds are not fully healed. Further studies should include longer trial times that include late scar formation.

In summary, we were able to describe two metal concentration profiles. The first profile included zinc, iron, and magnesium, which reached their maximums during the proliferation phase. The second profile is copper, which shortly after the beginning of the proliferation phase decreased drastically. It is notable that, apart from magnesium, none of the metals we quantified reached the healthy skin concentration levels after 14 days. The differences between the kinetics measured by Lansdown et Al. [13] and our group may result from the wound type, and highlights the necessity of additional research in the field of wound healing to describe the specific kinetics for different types of wounds. Further research to establish long-term comparisons between fully stabilized scar and healthy skin should be considered, as there is no literature available about metal content of scar tissue. Moreover, refined wound content analysis, narrowed to specific regions, should be conducted in order to eliminate healthy skin bias during their determination.

## Conclusion

We established and refined the kinetics of metal concentrations during the healing of open wounds in a rat model. This study offers new perspectives in the field of wound treatment, as healing might be enhanced by time-specific supplementation of iron, copper, zinc, and magnesium. According to our results, the supplementation of zinc, iron, and magnesium should be considered from the late inflammation until the mid-proliferation phase. Copper supplementation, on the other hand, may be restricted to the early stage of the wound healing. This work may be a first step into understanding the requirements of different elements during various wound healing phases, which may help to optimize compositions of metal ion-based ointments and wound coverages to enhance the wound healing process.

## Electronic Supplementary Material

Supplement Figure 1 Hematoxylin and eosin staining of the median slide of the rat wounds at time (A) 8 h; (B) 16 h; (C) 1 d; (D) 2 d; (E) 3 d; (F) 5 d; (G) 7 d; (H) 10 d; (I) 14 d. Black arrows point the wound edges; red arrows point epidermis extremities. H&E magnification, 2x (Keyence BioZero) (PNG 9222 kb)

High Resolution Image (TIF 22315 kb)

Supplement Figure 2 Ki-67 immunofluorescence (red), autofluorescence (green) and DAPI (blue) staining of (A) healthy rat skin; margin of the rat wound at time (B) 3 d; (C) 5 d; (D) 7 d. White asterisks: healthy dermis; red asterisks; granulation tissue; arrows: epidermis; white arrow: Ki-67 positive keratinocyte; green arrow: Ki-67 negative keratinocyte. Fluorescence microscopy magnification 10x (Zeiss AxioVert 200 m with ApoTome) (PNG 13536 kb)

High Resolution Image (TIF 54010 kb)
