# Supplementary figures and images for: Tissue Concentrations of Zinc, Iron, Copper, and Magnesium During the Phases of Full Thickness Wound Healing in a Rodent Model
Source: Biol Trace Elem Res. 2018 Dec 14;191(1):167–76. doi: 10.1007/s12011-018-1600-y (PMC6656798; doi:10.1007/s12011-018-1600-y)

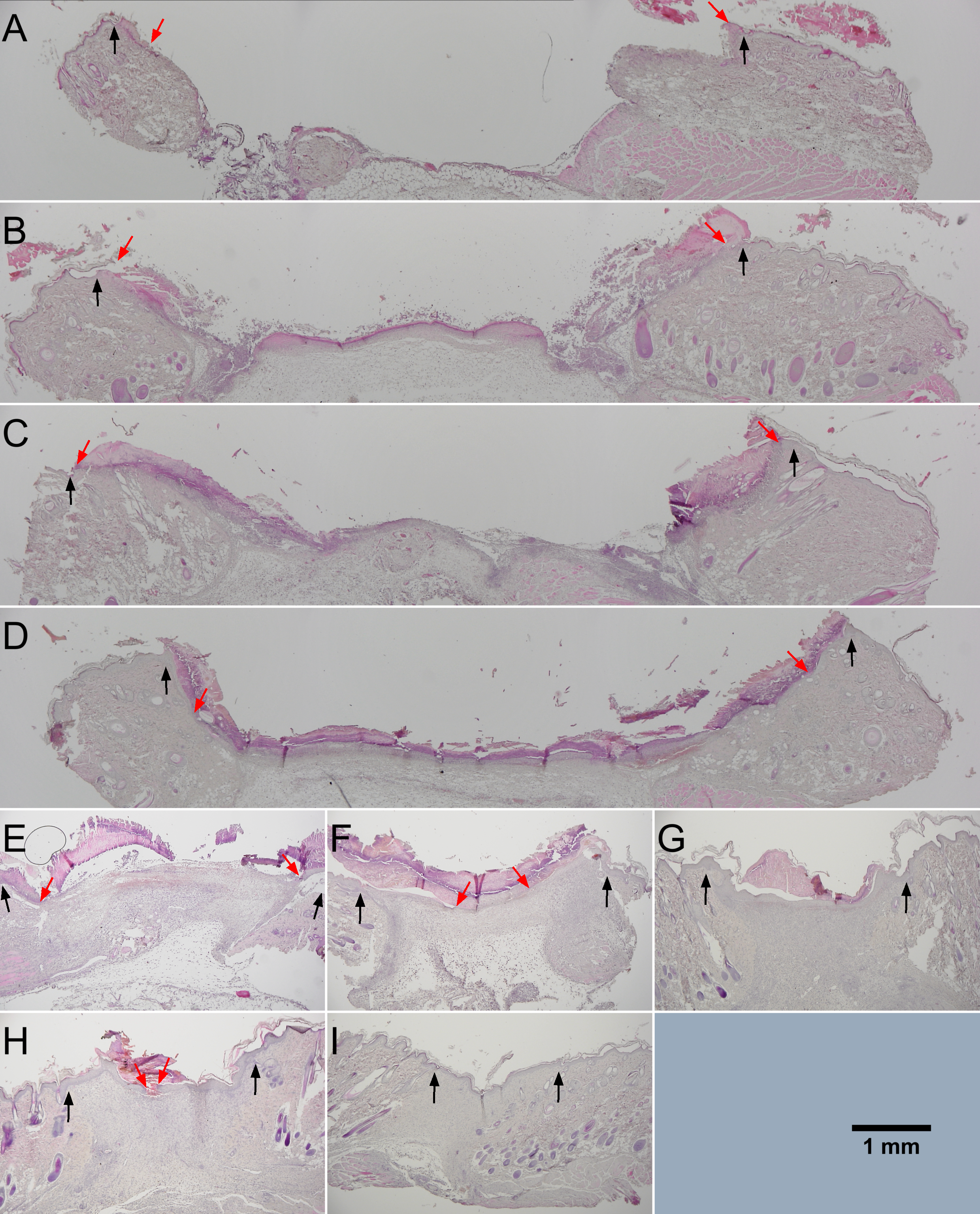

Supplement: Supplementary file 1 — Hematoxylin and eosin staining of the median slide of the rat wounds at time (A) 8 h; (B) 16 h; (C) 1 d; (D) 2 d; (E) 3 d; (F) 5 d; (G) 7 d; (H) 10 d; (I) 14 d. Black arrows point the wound edges; red arrows point epidermis extremities. H&E magnification, 2x (Keyence BioZero) (PNG 9222 kb) [file 12011_2018_1600_Fig9_ESM.png]

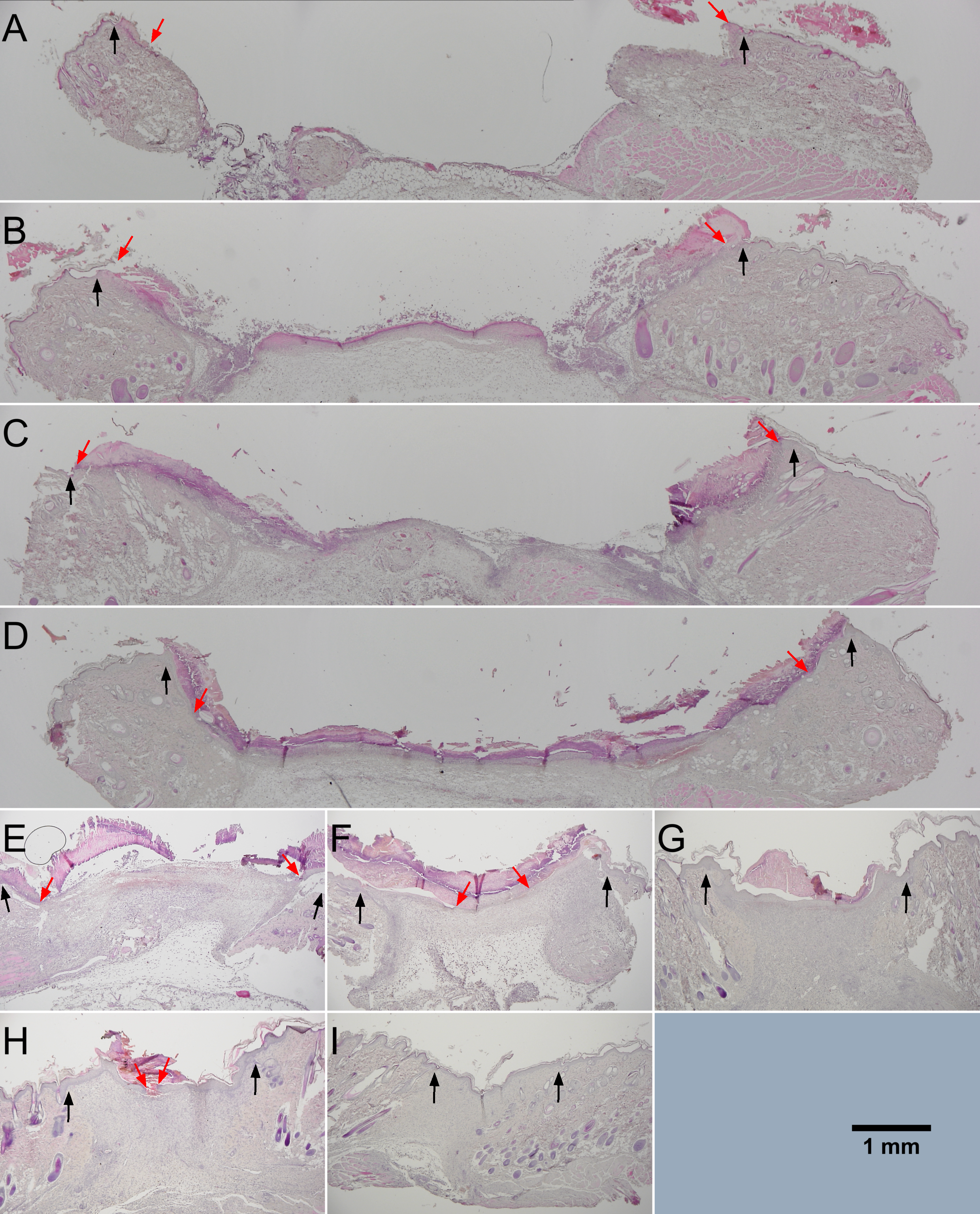

Supplement: Supplementary file 2 — High Resolution Image (TIF 22315 kb) [file 12011_2018_1600_MOESM1_ESM.tif]

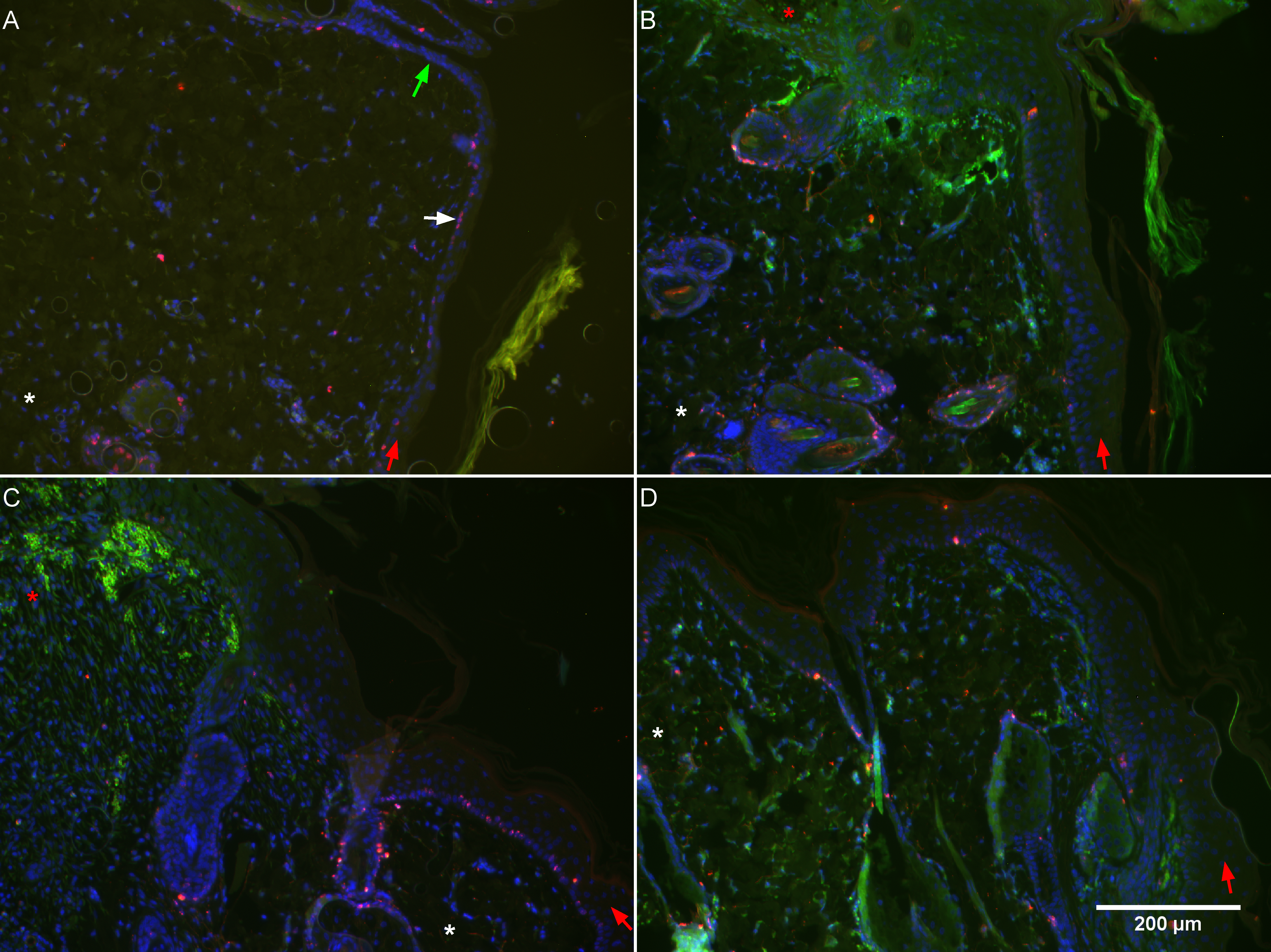

Supplement: Supplementary file 3 — Ki-67 immunofluorescence (red), autofluorescence (green) and DAPI (blue) staining of (A) healthy rat skin; margin of the rat wound at time (B) 3 d; (C) 5 d; (D) 7 d. White asterisks: healthy dermis; red asterisks; granulation tissue; arrows: epidermis; white arrow: Ki-67 positive keratinocyte; green arrow: Ki-67 negative keratinocyte. Fluorescence microscopy magnification 10x (Zeiss AxioVert 200 m with ApoTome) (PNG 13536 kb) [file 12011_2018_1600_Fig10_ESM.png]

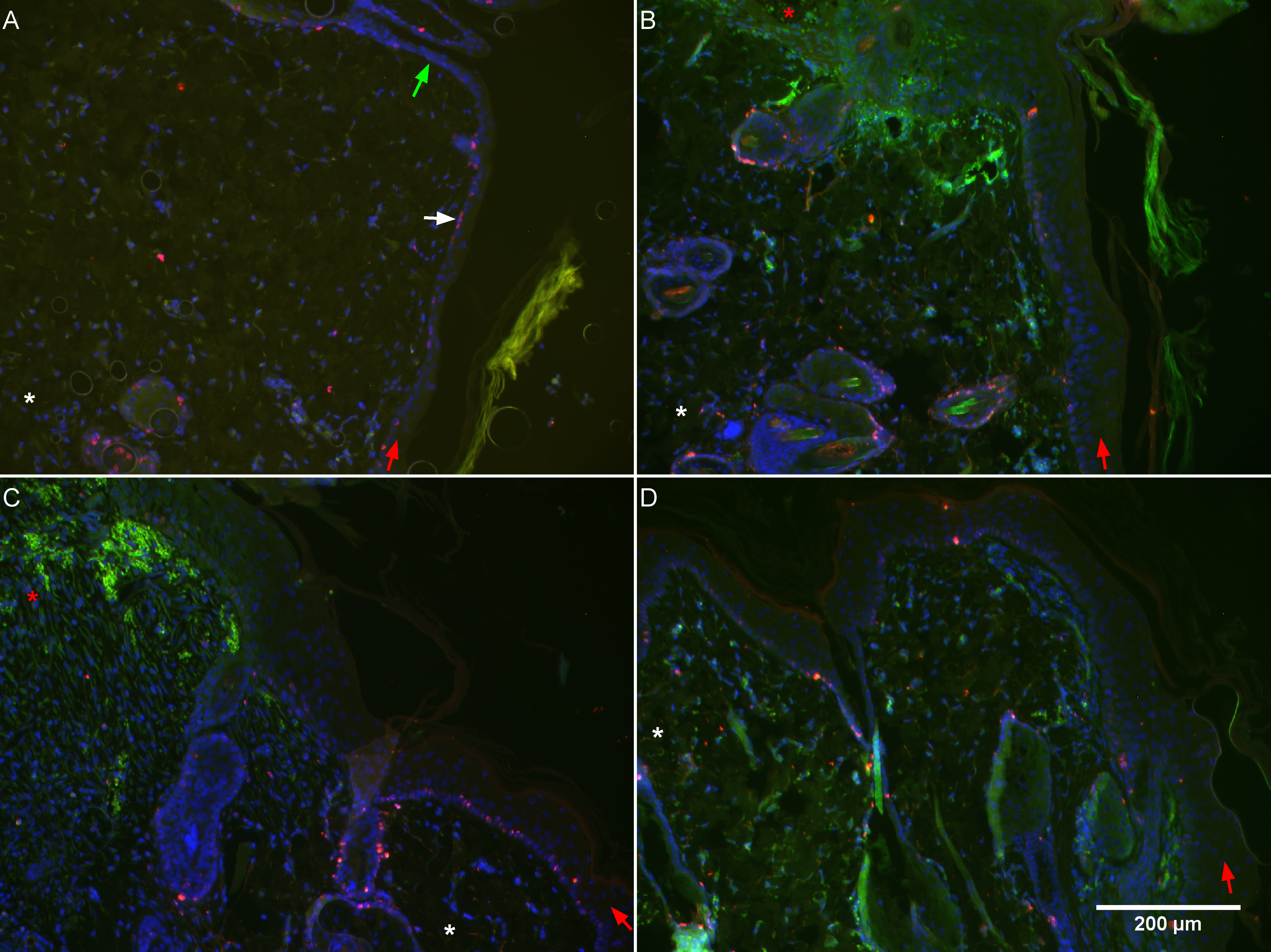

Supplement: Supplementary file 4 — High Resolution Image (TIF 54010 kb) [file 12011_2018_1600_MOESM2_ESM.tif]
